# Supplementary material for: Intermittent hypoxia in neonatal rodents affects facial bone growth
Source: PLoS One. 2023 Oct 11;18(10):e0282937. doi: 10.1371/journal.pone.0282937 (PMC10566710; doi:10.1371/journal.pone.0282937)
Supplement: S1 Data — (DOCX) [file pone.0282937.s001.docx]

**Body Weight (g)**

3W-Male

| Control | IH |
| --- | --- |
| 34.9  37.4  36  37.4  48  51  48.5 | 36.8  31  36.7 |

3W-Female

| Control | IH |
| --- | --- |
| 31.9  49.5  44.5  46.4 | 30  36.7  42.5  52  37.5  44.1 |

4W-Male

| Control | IH |
| --- | --- |
| 82.6  69.8  71.8  70.4  78.5  83.7  81.1 | 81.6  83  76.2  75.3  70.1  78.2  70.6 |

4W-Female

| Control | IH |
| --- | --- |
| 62.6  76.6  64.4  76.1  58.4  64.3 | 74.3  77.5  57.3  73.8 |

5W-Male

| Control | IH |
| --- | --- |
| 97.7  100.2  102  104.3  106.4 | 120.3  110  116.7  122.5  110  113.8  118  119.5 |

5W-Female

| Control | IH |
| --- | --- |
| 96.6  91.7  83.7  86.5  91.2  96.2  91.7  98.9 | 104.1  102.1  112.5 |

**Dry Mandible (g)**

3W-Male

| **Control** | **IH** |
| --- | --- |
| 0.15 | 0.16 |
| 0.18 | 0.16 |
| 0.15 | 0.17 |
| 0.17 | 0.14 |
| 0.18 | 0.16 |
| 0.16 |  |
| 0.16 |  |

3W-Female

| **Control** | **IH** |
| --- | --- |
| 0.14 | 0.15 |
| 0.14 | 0.15 |
| 0.13 | 0.16 |
| 0.15 | 0.14 |
| 0.16 | 0.15 |
| 0.15 |  |

4W-Male

| **Control** | **IH** |
| --- | --- |
| 0.21 | 0.2 |
| 0.22 | 0.21 |
| 0.23 | 0.2 |
| 0.21 | 0.21 |
| 0.21 | 0.2 |
| 0.2 | 0.21 |
| 0.22 | 0.18 |
|  |  |

4W-Female

| **Control** | **IH** |
| --- | --- |
| 0.21 | 0.21 |
| 0.2 | 0.18 |
| 0.2 | 0.21 |
| 0.2 | 0.21 |
| 0.21 |  |
| 0.24 |  |

5W-Male

| **Control** | **IH** |
| --- | --- |
| 0.27 | 0.3 |
| 0.28 | 0.3 |
| 0.27 | 0.3 |
|  | 0.29 |
|  | 0.33 |
|  | 0.3 |
|  |  |

5W-Female

| **Control** | **IH** |
| --- | --- |
| 0.25 | 0.3 |
| 0.26 | 0.28 |
| 0.28 | 0.3 |
| 0.25 |  |
| 0.28 |  |

**Norepinephrine (pg/mL)**

3W-Male

| Control  19.8  37.7  36.4  26.1 | IH  35.9  41.3  37.3  40  36.1  41.8 |
| --- | --- |

3W-Female

| Control  22.2  17.2  19.5  12.65  29.83  29.54  37.38  33.91  10.11  20.77 | IH  35.9  26.3  21.9  27.81  37.3  41.04  48.79 |
| --- | --- |

4W-Male

| Control | IH |
| --- | --- |
| 35.616 | 42.149 |
| 38.257 | 44.644 |
| 36.365 | 39.617 |
| 36.317 | 44.831 |
| 37.092 | 41.629 |
| 34.552 | 37.808 |
| 38.691 |  |
|  |  |

4W-Female

| Control | IH |
| --- | --- |
| 33.182 | 32.434 |
| 34.144 | 24.512 |
| 30.914 | 39.406 |
| 34.917 | 34.592 |
|  |  |

5W-Male

| Control | IH |
| --- | --- |
| 35.99 | 42.732 |
| 35.77 | 39.611 |
| 35.18 | 42.648 |
|  | 40.185 |
|  | 42.137 |
|  | 46.65 |
|  | 48.934 |
|  |  |
|  |  |

5W-Female

| Control | IH |
| --- | --- |
| 41.77 | 36.359 |
| 41.796 | 38.999 |
| 42.726 | 44.177 |
| 40.073 |  |
| 42.272 |  |
| 39.042 |  |
|  |  |

Linear Measurements Summary (mm)

| Males | 3W | 4W | 5W | 3W-SE | 4W-SE | 5W-SE |
| --- | --- | --- | --- | --- | --- | --- |
| Intermolar Width-C | 6.39 | 6.23 | 6.45 | 0.053 | 0.142 | 0.115 |
| Intermolar Width-IH | 6.17 | 6.22 | 6.37 | 0.056 | 0.104 | 0.037 |
| Palatal Width-C | 4.24 | 4.3 | 4.24 | 0.027 | 0.056 | 0.051 |
| Palatal Width-IH | 4.15 | 4.28 | 4.54 | 0.043 | 0.04 | 0.077 |
| Inter-Zygo-C | 10.11 | 10.82 | 11.13 | 0.128 | 0.135 | 0.151 |
| Inter-Zygo-IH | 10.22 | 10.42 | 11.35 | 0.242 | 0.051 | 0.477 |
| Intercondylar-C | 15.74 | 14.73 | 15.8 | 0.451 | 0.157 | 0.374 |
| Intercondylar-IH | 14.44 | 13.77 | 16.03 | 0.125 | 0.157 | 0.219 |
| Mn-Length-C | 18.33 | 20.06 | 20.71 | 0.255 | 0.249 | 0.104 |
| Mn-Length-IH | 18.73 | 19.33 | 21.81 | 0.126 | 0.152 | 0.054 |
| Mn-Height-C | 8.4 | 9.85 | 10.34 | 0.117 | 0.09 | 0.184 |
| Mn-Height-IH | 8.57 | 9.53 | 10.77 | 0.168 | 0.079 | 0.051 |
|  |  |  |  |  |  |  |
| Female | 3W | 4W | 5W | 3W-SE | 4W-SE | 5W-SE |
| Intermolar Width-C | 6.45 | 6.44 | 6.23 | 0.103 | 0.074 | 0.107 |
| Intermolar Width-IH | 6.12 | 6.17 | 6.55 | 0.127 | 0.057 | 0.078 |
| Palatal Width-C | 4.2 | 4.05 | 4.15 | 0.041 | 0.063 | 0.063 |
| Palatal Width-IH | 4.15 | 4.23 | 4.66 | 0.023 | 0.05 | 0.069 |
| Inter-Zygo-C | 10.28 | 10.46 | 10.69 | 0.098 | 0.152 | 0.187 |
| Inter-Zygo-IH | 9.96 | 10.7 | 11.48 | 0.051 | 0.292 | 0.056 |
| Intercondylar-C | 15.88 | 15.24 | 15.35 | 0.295 | 0.191 | 0.303 |
| Intercondylar-IH | 13.78 | 14.41 | 15.69 | 0.105 | 0.317 | 0.228 |
| Mn-Length-C | 18.62 | 18.72 | 20.54 | 0.136 | 0.276 | 0.2 |
| Mn-Length-IH | 18.85 | 19.06 | 21.38 | 0.105 | 0.378 | 0.269 |
| Mn-Height-C | 8.54 | 9.09 | 10.04 | 0.169 | 0.161 | 0.13 |
| Mn-Height-IH | 8.65 | 9.41 | 10.49 | 0.053 | 0.179 | 0.14 |
