# Supplementary material for: Intermittent hypoxia in neonatal rodents affects facial bone growth
Source: PLoS One. 2023 Oct 11;18(10):e0282937. doi: 10.1371/journal.pone.0282937 (PMC10566710; doi:10.1371/journal.pone.0282937)
Supplement: Attachment — Submitted filename: Response to reviewers.docx [file pone.0282937.s002.docx]

**Response to Reviewers in bold with underline**

Reviewers' comments:

Reviewer's Responses to Questions

**Comments to the Author**

1. Is the manuscript technically sound, and do the data support the conclusions?

The manuscript must describe a technically sound piece of scientific research with data that supports the conclusions. Experiments must have been conducted rigorously, with appropriate controls, replication, and sample sizes. The conclusions must be drawn appropriately based on the data presented.

Reviewer #1: Yes

2. Has the statistical analysis been performed appropriately and rigorously?

Reviewer #1: Yes

3. Have the authors made all data underlying the findings in their manuscript fully available?

**Response**: We addressed this issue as guided.

The [PLOS Data policy](http://www.plosone.org/static/policies.action#sharing) requires authors to make all data underlying the findings described in their manuscript fully available without restriction, with rare exception (please refer to the Data Availability Statement in the manuscript PDF file). The data should be provided as part of the manuscript or its supporting information, or deposited to a public repository. For example, in addition to summary statistics, the data points behind means, medians and variance measures should be available. If there are restrictions on publicly sharing data—e.g. participant privacy or use of data from a third party—those must be specified.

Reviewer #1: Yes

4. Is the manuscript presented in an intelligible fashion and written in standard English?

PLOS ONE does not copyedit accepted manuscripts, so the language in submitted articles must be clear, correct, and unambiguous. Any typographical or grammatical errors should be corrected at revision, so please note any specific errors here.

Reviewer #1: Yes

5. Review Comments to the Author

Please use the space provided to explain your answers to the questions above. You may also include additional comments for the author, including concerns about dual publication, research ethics, or publication ethics. (Please upload your review as an attachment if it exceeds 20,000 characters)

Reviewer #1: It is a very interesting and rigorous experiment. I did some suggestions. Please see below

Intermittent Hypoxia in Neonatal Rodents Affects Facial Bone Growth

Abstract: Without comments

Introduction: Without comments

Methods:
• How were selected the control and the experimental groups? Please explain it on the experimental design section. You mentioned age and sex matched on abstract, results and conclusions but not on methods section.

**Response:** Because we purchased even numbers of pregnant rats each order, we could designate Treated vs. Control groups by flipping a coin. Then, after pups are born, we matched the numbers of pups in each group by sacrificing pups from a larger cohort. We matched age by sacrificing both groups on the same day at the same time. We have revised the lines like the following:

**“We treated the pups from randomly-selected 7 mothers for 1 h because we found that 1 h treatment (or 7 cycles of IH exposure) reflects a clinically not unusual as well as sufficiently mild condition devoid of conspicuous neural damage. Randomly-selected offspring and 7 mothers of the control group were exposed to ambient air.”**

• Why all statistical inferences tests were two-tailed If your hypothesis assumed decreasing of mandibular bone quality and elevated norepinephrine levels. Then you assumed the direction of the difference. Please check.

**Response:** Even though we hypothesized bone quality and the blood levels of NE with a direction, one would not know until the results has revealed. In our case, one example was NE level changes in females and other example was IH influence on weights. Therefore, we need to be as conservative as possible when inference tests are performed.

Results: Without comments

Discussion:
• The following statement needs to be referenced: Although controversial, many previous malocclusion studies were based on the assumption that life-supporting equipment maintained in the oral cavity of preterm infants is the principal causal contributor to malocclusions

**Response:** References are added as recommended like the following.

**“Although controversial, many previous malocclusion studies were based on the assumption that life-supporting equipment maintained in the oral cavity of preterm infants is the principal causal contributor to malocclusions [38, PamuKu, 2020; 39, Kim et al., 2019].”**

Limitation of the study: Please include on the discussion

**Response:** When the study was initiated, we were reasonably comfortable with the hypothesis. One regret may be we should have included ‘a positive control group’ with pups treated with adrenergic agonists. We included the following in conclusion of the final version.

**“Unfortunately, we did not include a positive control group such as pups treated with an adrenergic agonist. However, we showed that the enhanced sympathetic drive from an IH challenge elicits transient metabolic changes, with significant effects on weight.”**

Conclusion: without comments

Ethics Approval: It is presented

References: There are some of them with more than 10 years published, please consider the possibility to update

6. PLOS authors have the option to publish the peer review history of their article ([what does this mean?](https://journals.plos.org/plosone/s/editorial-and-peer-review-process#loc-peer-review-history)). If published, this will include your full peer review and any attached files.

If you choose “no”, your identity will remain anonymous but your review may still be made public.

**Do you want your identity to be public for this peer review?** For information about this choice, including consent withdrawal, please see our [Privacy Policy](https://www.plos.org/privacy-policy).

Reviewer #1: **Yes:** Angela Fernanda Espinosa Aranzales

[NOTE: If reviewer comments were submitted as an attachment file, they will be attached to this email and accessible via the submission site. Please log into your account, locate the manuscript record, and check for the action link "View Attachments". If this link does not appear, there are no attachment files.]

While revising your submission, please upload your figure files to the Preflight Analysis and Conversion Engine (PACE) digital diagnostic tool, <https://pacev2.apexcovantage.com/>. PACE helps ensure that figures meet PLOS requirements. To use PACE, you must first register as a user. Registration is free. Then, login and navigate to the UPLOAD tab, where you will find detailed instructions on how to use the tool. If you encounter any issues or have any questions when using PACE, please email PLOS at [figures@plos.org](mailto:figures@plos.org). Please note that Supporting Information files do not need this step.

*In compliance with data protection regulations, you may request that we remove your personal registration details at any time.* [*(Remove my information/details)*](https://www.editorialmanager.com/pone/login.asp?a=r)*. Please contact the publication office if you have any questions.*
